# Can MRI predict return to sport after anterior cruciate ligament reconstruction? A systematic review of the literature

**DOI:** 10.1007/s11547-025-01973-5

**Published:** 2025-03-10

**Authors:** Riccardo D’Ambrosi, Luca Maria Sconfienza, Domenico Albano, Amit Meena, Elisabeth Abermann, Christian Fink

**Affiliations:** 1 IRCCS Istituto Ortopedico Galeazzi, Milan, Italy; 2 Dipartimento di Scienze Biomediche per la Salute, Università degli Studi di Milano, Milan, Italy; 3 Dipartimento di Scienze Biomediche, Chirurgiche ed Odontoiatriche, Università degli Studi di Milano, 20122 Milan, Italy; 4 Department of Orthopaedics, Shalby Hospital, Jaipur, India; 5 Gelenkpunkt-Sports and Joint Surgery FIFA Medical Centre of Excellence, Innsbruck, Austria; 6 Research Unit for Orthopaedic Sports Medicine and Injury Prevention (OSMI), Private University for Health Sciences Medical Informatics and Technology, Innsbruck, Austria

**Keywords:** Anterior cruciate ligament, Return to sport, Magnetic resonance imaging, Graft maturation

## Abstract

**Purpose:**

To analyze whether magnetic resonance imaging (MRI) can predict return to sport after anterior cruciate ligament (ACL) reconstruction and whether a correlation exists between return to sports, level of activity and MRI signals.

**Methods:**

The search terms selected for inclusion in the title, abstract, and keyword fields were as follows: ‘anterior cruciate ligament’ OR ‘ACL’ AND ‘graft maturation’ OR ‘MRI’ AND ‘return to sport’ OR ‘sports activity.’ For each study, patient data and the MRI protocol used to assess graft maturation were extracted. An analysis of the correlations between MRI and ACL reconstruction was performed.

**Results:**

A total of 394 patients were included from 7 studies. The mean radiological follow-up was 19.06 ± 11.02 months. Three studies reported no correlations between graft bending angle, signal/noise ratio, signal intensity or Howell score and return to sport. One study revealed that T2* was correlated with return to sport. A further investigation demonstrated that those who were able to regain their preinjury athletic performance exhibited considerably lower ACL/PCL ratio and ACL/muscle ratio of the ACL mid-substance compared to those who were unable to attain the same level of athletic performance. Only one study reported correlations between 12-month SNRs and 60-month Cincinnati, Lysholm and Tegner activity scales, whereas Biercevicz revealed that the combination of volume and the SI predicted the KOOS score at the 5-year follow-up.

**Conclusions:**

There are no reliable radiological parameters available that correlate with return to sport after anterior cruciate ligament reconstruction, but MRI can potentially play a key role in closing this gap.

**Level of evidence:**

Systematic review of level IV.

**Study registration:**

PROSPERO—CRD42024574365.

## Introduction

The annual incidence of anterior cruciate ligament (ACL) reconstruction in the USA has increased across all age cohorts, with the most significant increases observed in patients under 15 years of age and those over 40 years of age [1, 2]. In 1994, the incidence rate of ACL reconstruction shown an upward trend, rising from 32.9 per 100,000 person-years to 68.6 per 100,000 person-years by the year 2016 [1, 2]. Recent estimates indicate that approximately 200,000 ACL reconstructions are carried out in the USA each year [3]. Due to a burgeoning population and a rising trend toward physical activity and fitness, the incidence of ACL injuries and subsequent reconstructions is expected to continue to increase [4]. However, despite the annual increase in the number of ACL reconstructions conducted, the recurrence and ineffectiveness of the graft continue to pose significant obstacles [5]. The rerupture rate mentioned in the literature varies from 0 to 25%. However, larger and more extensive studies indicate rates ranging from 2 to 7.7%. Although other factors can contribute to this, one possible reason for ACL reconstruction failure is the resumption of physical activity before the graft has fully healed and integrated [4, 5].

At present, there is no agreement on the optimal measures to be employed in determining an athlete's readiness to resume sports activities. Most methods primarily rely on the duration after surgery and/or utilize tangible milestones as a general reference [6]. Regrettably, most examinations focus primarily on evaluating the muscles surrounding the knee joint rather than the strength and integrity of the healing ACL graft [7]. Research has demonstrated that there is a greater likelihood of experiencing a second ACL tear during the initial 12 months following initial reconstruction. This risk is associated with either a failure or tear of the graft in the same knee or a tear of the ACL in the opposite knee [8]. Magnetic resonance imaging (MRI) is the method of choice for assessing soft tissues around joints. MRI can evaluate tissue quality by measuring normalized signal intensity, where lower signal intensity indicates tissues similar to normal tendons and ligaments [9]. Additionally, MRI can measure the normalized graft volume or cross-sectional area, which can help reestablish the mechanical qualities of the healing tissue [10]. Specifically, there is a correlation between the linear combination of graft volume and mean signal intensity and the in situ structural parameters. The signal intensity directly measures the amount of water present in the graft, which correlates with the organization of collagen, and, therefore, the tensile strength of the graft [11]. Research has demonstrated that greater blood vessel formation and water content during the initial stages of graft healing lead to greater signal intensity, which is inversely related to tensile strength. As the graft develops, the signal intensity subsequently diminishes. The normalized graft volume refers to the amount of tissue, which is also associated with the overall strength of the healing graft [12].

The aim of the present systematic review was to analyze whether MRI can predict return to sport after anterior cruciate ligament reconstruction and whether a correlation exists between return to sports, level of activity and MRI signals.

## Material and methods

The present study adheres to the Preferred Reporting Items for Systematic Reviews and Meta-Analyses (PRISMA) standards and has been duly registered in the PROSPERO Registry CRD42024574365 [13, 14]. The researchers employed the Measurement Tool to Assess Systematic Reviews (AMSTAR) 2 checklist in order to validate the quality of the scientific review. No ethical committee approval was needed for the present study, as no patients were involved [15].

### Research methodology

Case series, randomized controlled trials, controlled clinical trials (nonrandomized), prospective and retrospective comparative cohort studies, and case–control studies were included. Case reports and case series that did not provide correlations between the maturation of MRI grafts and the return to sports were excluded.

### Participants

Patients who had undergone ACL reconstructions and where postoperative MRI was used to investigate correlations between graft maturation and return to sports were the subjects of the investigations. Skeletally mature patients were classified as patients who had a closed physis on MRI.

### Interventions

The studies were incorporated if they reported correlations between MRI signals and return to sport after anterior cruciate ligament surgery, irrespective of the surgical technique or graft employed. ACL repair or revision surgery was regarded as an exclusionary criterion.

### Types of outcome measures

The outcome measures extracted from the studies were the correlations between postoperative MRI and return to sport. No subanalyses were performed according to concomitant procedures. Data regarding MRI protocols were reported for each study.

### Follow‐up

Studies with a minimum follow-up period of six months were included. Studies that reported prolonged average follow-up times but did not specify a minimum follow-up were excluded unless it was feasible to identify and isolate data from the subgroup with more than six months of follow-up data. Consequently, all patients who were evaluated underwent a minimum of six months of follow-up.

### Information sources and search

A comprehensive search was conducted in the PubMed (MEDLINE), Scopus, EMBASE, and Cochrane Library databases to identify pertinent literature. We conducted a search in July 2024 that encompassed all English-language research published between January 1990 and July 2024. The search was conducted and validated with the assistance of two independent reviewers (R.D. and E.A.). The title, abstract, and keyword fields were populated with the following search terms and their expansions: "anterior cruciate ligament" or "ACL" AND "graft maturation" or "magnetic resonance imaging" AND "return to sport" or "sports activity." Ultimately, only manuscripts that were published in English were considered.

### Data collection and analysis

#### Study selection

The titles of the articles that were retrieved were examined, and if applicable, the abstracts were read in addition. The entire content of the remaining articles was assessed for eligibility after studies that did not satisfy the eligibility criteria were excluded. The authors conducted a comprehensive review and discussion of all the selected articles, references, and articles that were excluded from the study in order to reduce the risk of bias. The principal investigator was responsible for making the final decision in the event of any disagreements between the reviewers. The reference lists of the included studies and relevant systematic reviews were manually evaluated at the conclusion of the process to identify any supplementary studies that may have been overlooked during the initial search.

#### Data collection process

The initial two authors utilized a computerized tool developed with Microsoft Access (Version 2010, Microsoft Corp.) to extract data from the selected articles and compile the information. First author conducted an additional validation of each article prior to its analysis. Patient data (age, sex) and the MRI protocol employed to evaluate graft maturation were extracted for each study.

#### Level of evidence

The level of evidence was categorized using the Oxford Levels of Evidence established by the Oxford Centre for Evidence-Based Medicine [17].

#### Evaluation of the quality of studies

The Methodological Index for Nonrandomized Studies (MINORS) score was employed to assess the quality of the selected studies. The inventory consists of 12 items, the final four of which are tailored to comparative studies. A tally of 0–2 points was assigned to each item. For comparative studies, the optimal score was 24 points, while for noncomparative studies, it was 16 [18]. Additionally, the ROBINS-I instrument was implemented to evaluate each article in accordance with the AMSTAR-2 guidelines [15].

### Statistical analysis

The mean ± standard deviations (SDs) of the extracted quantitative parameters (age, follow-up time, and PROM results) are presented in the articles. Otherwise, alternative values, such as medians or ranges, were extracted. The results could not be compared in a meta-analysis due to the significant statistical and methodological heterogeneity of the included studies. Rather, a narrative description and comparison of the correlations between the radiological score and return to sport were conducted.

## Results

### Search results

**Fig. 1:**
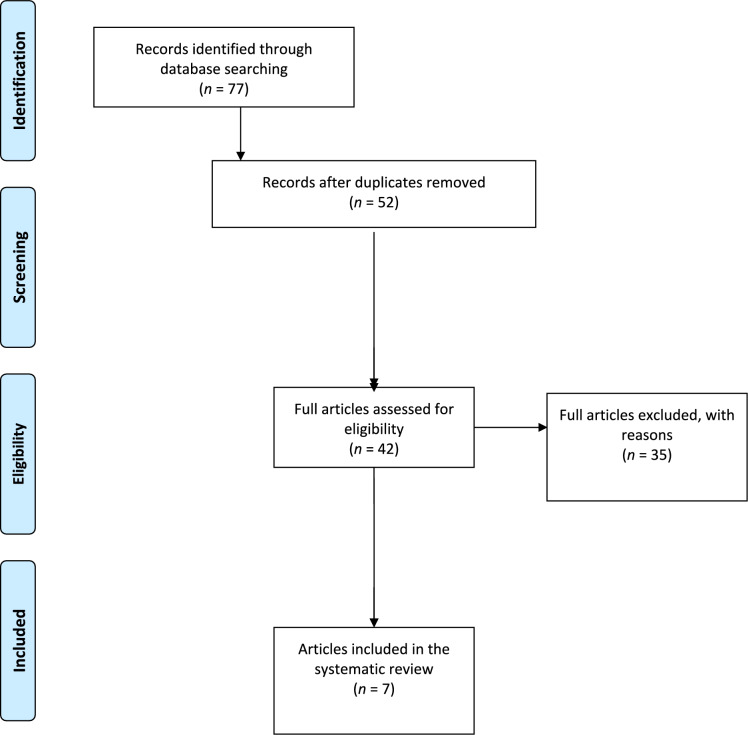
A flowchart of the articles screening performed in this study

A total of 77 studies were identified through the electronic search. Following the removal of 15 duplications, 52 studies were left. Following the abstract review, 10 of these studies were excluded, resulting in 42 studies. An additional 35 articles were excluded in accordance with the inclusion and exclusion criteria. After doing a manual search of the reference lists of the selected papers, no further investigations were identified. A total of seven studies remained for examination [19–25]. The flowchart of the study selection procedure is depicted in Fig. 1. The examined studies exhibited a mean MINORS score of 13.7, hence substantiating the methodological rigor of the existing literature (Table 1). Of the 7 articles included in the review, 6 had a Grade III level of evidence [19–21, 23–25], and one had a Grade I level of evidence [22].

**Table 1:** Surgical details of the studies included in the systematic review

Author and Year	Level of Evidence	MINORS	Patients (M/F)	Age	Graft	Surgical Technique	Follow-up (maximum)
Li et al., 2019 [19]	3	13	43 (37/6)	30 (18–40)	Hamstring	Anteromedial	12 months
Zhou et al. 2024 [20]	3	19	82 Return to sport group: 53 (42/11) Non-return to sport group: 29(22/7)	Return to sport group: 27.65 ± 6.04 Non-return to sport group: 28.25 ± 6.45	Hamstring	Anteromedial	Return to sport group: 9.5 ± 0.37 Non-return to sport group: 9.43 ± 0.52
Lutz et al. 2021 [21]	3	12	17 (17/0)	28.3 ± 7.0	Hamstring	Anteromedial	24 months
Cusumano et al. 2022 [22]	1	18	49 Autograft: 21/3 Allograft: 23/2	Autograft: 27 ± 7 Allograft: 30 ± 6	Autograft: Hamstring (24) Allograft: Peroneal (17) or posterior tibialis (8)	Transtibial	12 months (radiologic) 60 months (clinical)
Bouguennec et al. 2021 [23]	3	12	149 (95/54)	36.4	Hamstring	Outside-in	25.7 months
Biercevicz et al. 2015 [24]	3	11	16 (6/10)	24 ± 10	BPTB (11) or Hamstring (5)	Transtibial	5 years
Li et al. 2017 [25]	3	11	38	30.89	Hamstring (21) or tibialis anterior allograft (17)	Anteromedial	12 months

BPTB = Bone-Patellar Tendon-Bone

### Demographic data

A total of 394 patients were included, of which 263 (66.75%) were male, 93 (23.60%) were female, and 38 (9.65%) cases did not report sex. The mean age at surgery was 31.51 ± 4.04 years. The mean radiological follow-up was 19.06 ± 11.02 months (Table 2).

**Table 2:** Correlations between radiological and return to sports outcomes after anterior cruciate ligament reconstruction

Author	MRI Protocol	Radiologic Parameters	Clinical Score	Correlations
Li et al., 2019 [19]	3.0-T MRI Sagittal images with oblique PD–fat saturation 3- dimensional dual-echo steady-state imaging or 3D PD	SNR: 19.4 (2.0—46.0) GBA:55.8° (40.6°-68.7°)	Lysholm Tegner	GBA was positively correlated with SNR No correlations with return to sport score
Zhou et al. 2024 [20]	3.0-T MRI using PD TSE and T2 mapping	SNR Return to sport: 12.37 ± 2.74* Non-return to sport: 15.07 ± 3.32* T2* values for intra-articular ACL graft tissues Return to sport: 17.69 ± 2.48* Non-return to sport: 14.92 ± 2.28*	1 Postoperative time of > 9 months; 2. Limb symmetry > 90% on 3 tests (single hop, triple hop, and triple hop crossover); 3. IKDC 2000 subjective score of > 90; 4. ACL-RSI score of > 56	Spearman correlation coefficient of T2* value with RTS was 20.41 (P = .02). The ROC analysis of T2* for predicting failure to RTS obtained under the curve of 0.79 (95% CI, 0.75–0.83). The ROC curve analysis revealed that a cutoff of 16.65 for T2* yielded a sensitivity of 67.9% and specificity of 88.2% for predicting failure to RTS
Lutz et al. 2021 [21]	3.0 Tesla whole-body Dedicated 8-channel knee coil to assess graft maturation process On sagittal PD intermediate-weighted images parallel to ACL	APR: Proximal: 2.4 Mid-substance: 2.1 Distal: 2.0 AMR: Proximal: 0.48 Mid-substance: 0.41 Distal: 0.4	IKDC 89 Lysholm 88 Tegner 6 65% (11/17) achieved the preinjury Tegner level	• APR and AMR were not correlated with any return to sports score • APR and AMR of the mid-substance were significantly lower hypo-intense (p < 0.05) in patients who were able return to the preinjury sports level compared to those who were not able the return to the same sports activity
Cusumano et al. 2022 [22]	1.5 Tesla scanner 10° of flexion	SNR: Autograft: 9.8 ± 7.1 Allograft: 10.4 ± 8.0	Autograft 12 months: Cincinnati: 92.2 ± 6.9 Tegner: 6 (3–9) Lysholm: 94.4 ± 7.3 60 months: Cincinnati: 95.1 ± 7.3 Tegner: 6 (3–8) Lysholm: 92.5 ± 6.0 ACL-RSI: 78.8 ± 23.7 Allograft 12 months: Cincinnati: 86.7 ± 10.7 Tegner: 7 (3–9) Lysholm: 94.7 ± 5.0 60 months: Cincinnati: 94.7 ± 5.7 Tegner: 7 (3–9) Lysholm: 89.9 ± 9.2 ACL-RSI: 87.5 ± 25.9	SNR at 6 months had no correlations with clinical score SNR at 12 months had significant correlations with 60 months Cincinnati (*p* = 0.003), Lysholm (*p* = 0.027) and Tegner (*p* = 0.018)
Bouguennec et al. 2021 [23]	1.5 T Thickness of 2 mm PD-weighted images were acquired with the spin echo technique	SNR 2.0 (-14.0 – 17) Howell grade 1.51 (1–4)	Return to sport: 57% same level 22% lower level 12% changed sport 11% did not return Lysholm 89.8 (52–100) Tegner 6.5 (2–10)	SNR and Howell showed no correlation between with clinical score SNR-Lysholm (p = 0.72 – Pearson Test) SNR-Tegner (p = 0.64 – Pearson Test)
Biercevicz et al. 2015 [24]	3.0 T 3-D T1-weighted FLASH sequence	Median graft signal intensity and volume	KOOS Sport	The combination of volume and Signal intensity predicted KOOS sport (p = 0.04)
Li et al. 2017 [25]	3.0 T relaxed extended position 1 h of rest before the MRI scan Sagittal images were obtained with oblique PD fat saturation	SI SNR: 18.6 ± 7.6	IKDC Lysholm Tegner	No correlations with sports activity and SNR or SI

^*^statistically significant difference

*ACL* anterior cruciate ligament, *PCL* posterior cruciate ligament, *ACL-RSI* The Anterior Cruciate Ligament-Return to Sport after Injury Scale, *MRI* magnetic resonance imaging, *SNR* signal/noise ratio, *GBA* graft bending angle, *APR* ACL/PCL ratio, *AMR* ACL/muscle ratio, *SI* signal intensity, *STIR* Sagittal short time inversion recovery, *RTS* return to sport, *PD* proton density, *ROI* region of interest, *IKDC* International Knee Documentation Committee, *KOOS* The Knee Injury and Osteoarthritis Outcome Score, *T* Tesla

#### Surgical details

Four studies reported the use of an anteromedial technique for ACL reconstruction [19–21, 25], two reported the use of the transtibial technique [22, 24], and only one reported an outside-in technique [23]. In 341 cases, the graft choice was autologous hamstring tendons; in 11 cases, it was autologous bone-patellar tendon-bone; and in 42 cases, it was allograft tendons, of which 17 were peroneal tendons, 8 were posterior tibialis tendons, and 17 were tibialis anterior tendons.

#### Radiologic parameters

Five studies used 3.0 Tesla (T) MRI to examine patients [19–21, 24, 25], while 2 studies used 1.5 T MRI [22, 24].

Graft maturation was assessed with the signal/noise ratio (SNR) in 5 studies [19, 20, 22, 23, 25], 2 studies evaluated signal intensity [24, 25], and one study evaluated graft maturation using the ACL/PCL ratio (APR) and ACL/muscle ratio (AMR) [21]. Other parameters used were the graft bending angle (GBA) [19], T2* values for intra-articular ACL graft tissues [20], Howell scores [23] and the volume of the graft [24].

#### Return to sports parameters

For return to sports, different parameters were used: 5 studies used the Lysholm score [19, 21, 22, 24, 25]; 5 studies used the Tegner activity scale [19, 21–23, 25]; and one study each used the IKDC [25], Cincinnati [22], ACL-RSI [22] and KOOS Sport scores [24]. The study by Zhou et al. [20] assessed return to sport as patients who met all of the following criteria to pass the return to sports (RTS) test:

1. Postoperative time > 9 months;
2. Limb symmetry index of > 90% in 3 hop tests (single, triple, and triple crossover);
3. International Knee Documentation Committee (IKDC) 2000 subjective score of > 90;
4. The ACL returned to Sport After Injury scale (ACL-RSI) score was > 56.

#### Correlations between MRI and return to sport

Three studies reported no correlations between GBA, SNR, signal intensity or Howell score and return to sport [19, 23, 25].

One study revealed that T2* was correlated with return to sport (p = 0.02); in particular, the ROC analysis of T2* for predicting failure to RTS revealed an area under the curve of 0.79 (95% CI, 0.75–0.83). The ROC curve analysis revealed that a cutoff of 16.65 for T2* yielded a sensitivity of 67.9% and specificity of 88.2% for predicting failure to RTS [20]. Another study revealed that the APR and AMR of the ACL mid-substance were significantly lower (hypo-intense) in patients who could return to the preinjury sports level than in those who did not achieve the same sports level (*p* < 0.05) [21]. Only one study reported that the 12-month SNR was significantly correlated with the 60-month Cincinnati score (*p* = 0.003, *r* = 0.442), Lysholm score (*p* = 0.027, *r* =  − 0.334) and Tegner activity scale score (*p* = 0.018, *r* =  − 0.357) [22]. In contrast, Biercevicz revealed that the combination of volume and the SI score predicted the KOOS sport score (R2 = 0.37, *p* = 0.048) at the 5-year follow-up [24].

## Discussion

Although this systematic review highlighted several potential radiologic parameters to detect correlations with return to sports after ACL reconstruction, no radiological score could be confirmed as accurate for assessing the return to sports after ACL reconstruction. The studies included in the analysis were found to be inadequately planned for their intended purpose. These studies exhibited limitations such as small sample numbers, nonconsecutive groups of patients, and improper statistical methods for quantifying the overall correlation between each radiological parameter and return to sport.

Despite the strong support of the scientific literature for the necessity of radiological parameters for understanding the return to sport after anterior cruciate ligament reconstruction (ACLR), there is still a lack of adequate research on this topic.

The evaluation of patient knee results after ACL surgery is often based on clinical and functional outcomes, as well as patient-reported outcome assessments. Nevertheless, these evaluations may lack the impartiality required to quantify nuanced alterations in tendon conditions, which is essential for assessing and tracking the advancement of graft healing [26–29].

MRI provides a noninvasive evaluation specific to the graft and may offer advantages over conventional measures of success. Quantitative assessments of the strength of the SI of grafts are commonly employed in clinical research to better understand the biological mechanisms involved in the healing of tendon grafts after ACL surgery. These methods have primarily focused on the signal intensity (SI) of proton density (PD)/T2-weighted MRIs, which is an indirect indicator of water content. This water content has been associated with graft vascularity. Nevertheless, the MRI signal is contingent upon hardware-specific variables, PD signal scaling factors, voxel volume, pulse sequence weighting, and the location of the patient within the scanner. These characteristics make it difficult to compare successive scans and patients. Many investigations have utilized SNR measurements to standardize the graft SI values within each image and reduce concerns regarding variability across scan sessions [30–32].

In their study conducted in 2020, van Gronigen et al. sought to conduct a comprehensive review of the existing literature pertaining to biopsies, MRI SNR, and clinical outcomes in the context of graft-maturity assessment following autograft ACLR, with the objective of identifying potential associations between these variables. Based on human biopsy research, the authors have found that the remodeling of the graft remains an ongoing process for up to one year following ACLR. At around 6 months, the MRI SNR reached its maximum value, subsequently exhibiting a slow decline. The variation in MRI techniques and the technical limitations employed in existing research limit the ability to accurately predict graft maturity and clinical and functional outcome measures using the MRI graft SNR following ACLR [33].

Lansdown et al. assessed the longitudinal progression of advanced quantitative imaging measurements of the ACL graft subsequent to reconstruction and investigated the correlations between the graft measurements and patient-reported outcome (PRO) measures. The longitudinal evaluation following ACL reconstruction resulted in a substantial decrease in the T1ρ and T2 relaxation periods of the ACL graft. The T1ρ and T2 values in autograft reconstructions were substantially lower than those in allografts at 24 months. However, there was a trend toward lower T1ρ and T2 values in autograft reconstructions at 36 months after reconstruction. These shortened relaxation times are indicative of a more organized collagen structure, as well as a higher proteoglycan content, which is inversely correlated with the T1ρ relaxation time. Furthermore, the KOOS sport subscores were substantially correlated with the 24-month postoperative imaging data [34].

The incidence rates of ACL injuries are 1 in 50 individuals for male athletes and 1 in 36 individuals for female athletes over the course of one season. The potential consequences of this injury for an athlete's career can be severe, as only 40% to 60% of athletes are able to resume sports competitions at the same level [1, 2]. Moreover, the probability of experiencing repeated damage to either the same or opposite ACL is 19.4% if the athlete resumes their sport 9 months after the surgical procedure, and this likelihood increases by a factor of 7 for those who return earlier [1, 2]. This concerning data has led to a need from various individuals involved in the field—including surgeons, practitioners, patients, and coaches—for accurate and reliable practices and protocols for returning to sports. This includes the use of testing or monitoring biomotor abilities [6].

Additionally, several psychological questionnaires have been employed to assess the preparedness of patients for engaging in sports. An ACL tear and subsequent surgery have a notable psychological impact on an athlete. The significance of psychological preparedness for resuming sports activities has been demonstrated to be highly crucial. The most frequently mentioned reason for not returning to preinjury activity is the fear of reinjury. Self-esteem levels, as measured by patient-reported outcome scales, have been found to be significantly correlated with patient performance on functional tests and rehabilitation. Research has shown that females tend to have a more pessimistic psychological perspective, resulting in a lower rate of return to sports than males do. Research has also demonstrated that younger patients who have weaker psychological readiness, as determined by surveys, are more likely to experience a second ACL rupture [35].

Barber-Westin et al. evaluated the rates of reinjury to either knee when these criteria are applied and assessed the objective functional criteria used to determine when patients can return to athletics postoperatively. In order to authorize participation in sports activities, three objective criteria were implemented. Lower extremity muscle strength was the most frequently observed parameter, followed by lower limb symmetry and knee examination parameters such as effusion and range of motion. Eight studies listed two criteria for readmission to sports, twelve studies listed one criterion, and one study recommended three criteria. In seven studies, the failure rates of the ACL reconstructions varied from 0 to 3%, from 4 to 6% in six studies, from 7 to 10% in four studies, and from 14 to 24% in four studies. 14 studies (67%) did not report any contralateral ACL injuries; the remaining 7 studies reported contralateral injuries in 2 to 15% of patients [36].

In this context, MRI can play a key role in determining when a return to sports is possible and helping surgeons and physiotherapists in decision-making. Notwithstanding its potential, this technology nevertheless has certain limits. One issue of concern pertains to the potential lack of reproducibility in signal intensity values while using different magnets. For instance, the quantification of signal intensity is constrained by its reliance on image acquisition, resulting in potential fluctuations influenced by MRI sequence settings and scanner manufacturer, hence leading to variability contingent upon the location of the scan.

Advanced MRI techniques offer the potential for reduced scan durations and decreased variability between magnets. T2* relaxometry is an MRI sequence that has the ability to achieve this. Postoperative MRI assessment using T2* relaxometry with shorter echo times provides a noninvasive approach to examine the development of the graft. The quantification of signal intensity is influenced by picture acquisition parameters and the manufacturer of the equipment, making the data specific to the protocol, magnet, and institution. This restricts its usefulness as a readily applicable criterion for determining when an individual can return to play [37].

Biercevicz et al. conducted a study in which they used MRI scans to assess the effects of bioenhanced ACL repair in a pig model. They specifically performed T2* mapping of the ligaments 52 weeks following the repair. Their research revealed that T2* values were highly indicative of the maximum load, yield load, and linear stiffness, exhibiting a correlation similar to that reported in earlier studies that utilized signal intensity. The authors concluded that this may serve as the initial stage in creating a uniform method to evaluate the healing of grafts, irrespective of the parameters of acquisition and the kind of magnet, at various institutions. A further study conducted by the same research team examined the correlation between T2* relaxometry and the semiquantitative histology of healing ACL repair tissue. Once again, a pig model was utilized and scanned after 52 weeks to acquire T2* relaxation values and measure the ligament volume [38].

The ligaments were subsequently subjected to histologic analysis via the sophisticated Ligament Maturity Index (LMI). The T2* and volume of healing ligaments were both found to be significant predictors of the total LMI score, as were the subscores for cells, collagen, and vessels. A lower T2* value and greater volume were related to better histology scores. This is a significant advancement in confirming the effectiveness of this imaging technique for assessing the recovery of ACL tissue [38].

One problem with the T2* relaxation time is that it is correlated with the degree of tissue healing and the organization of collagen fibrils, both of which can fluctuate over time. The ability to predict healing is contingent upon the overall organization of the tissue, such as mature ligaments or scar tissue, as indicated by the duration of T2* acquisition. According to a single study, the ACL volume with the shortest T2* relaxation time made the greatest contribution to the anticipated strength of the healing ligaments [37].

Beveridge et al. employed a porcine model to integrate brief and long T2* relaxation times at 6, 12, or 24 weeks. The objective of this method was to develop a more thorough comprehension of the structural characteristics of the healing ACL by distinguishing between organized (short T2* time) and disorganized (longer T2* time) components. The ultimate objective was to create a temporal linear predictive model that could accurately predict the healing process, irrespective of the stage of healing. The research demonstrated that the measured values of the structural properties closely matched the projected values of the structural properties, which were derived from a multiple linear regression model that considered both short and long T2* relaxation times during the healing period. This implies that in order to develop the most precise model of graft healing, it is imperative to account for both long and short T2* relaxation durations, as the healing ACL is composed of both organized and unstructured collagen that endures changes over time [39].

## Limitations

There are multiple limitations in this study. The present systematic review revealed significant heterogeneity among the studies examined in terms of the radiological and clinical scores used, follow-up time, surgical technique and graft used. Another limitation was the methodological rigor of the chosen studies. The majority of the research consisted of case series with a diverse variety of grafts or MRI protocols and lacked a control group. This significant methodological constraint emphasizes the necessity for additional meticulously planned prospective research and deeper exploration of the topic.

## Future directions

The results of this systematic review encourage more studies aimed at the correlation between return to sport and MRI after ACL reconstruction using T2* mapping. A longitudinal study might help in determining whether T2* mapping values could predict the return to sport over time and would provide additional evidence that these noninvasive techniques can be employed to longitudinally monitor ligament deterioration or healing. T2* mapping has been proposed as an alternative measure of ligament integrity due to its potential to more accurately represent the low water content and highly organized collagen structure. According to the theory, T2* is supposed to represent the degree of collagen fiber orientation and alignment, as well as the spin-to-spin interactions of protons bonded to collagen, by incorporating B0 inhomogeneity caused by paramagnetic and diamagnetic effects. As a result, T2* is frequently regarded as a surrogate marker for ligament integrity and water content, collagen matrix organization, fiber orientation, and fiber alignment. Furthermore, future *in vivo* studies need to evaluate patients with different ACL pathologies such as partial and complete tears as well as healed, intact, aged, degenerated, and inflammatory ligaments. Last, the use of diffusion-tensor imaging may further help to quantitatively evaluate ACL fiber integrity and maturation using fractional anisotropy measurement [40–42].

## Conclusions

There is no reliable radiological parameter available to correlate with return to sport after anterior cruciate ligament reconstruction, but MRI can potentially play a key role in closing this gap. The studies included in this review have a number of limitations, such as inadequate statistical methodologies for evaluating correlation performance, small sample sizes, nonconsecutive patient groups, and inappropriate study designs.

## Data Availability

Raw data are available upon request to the corresponding author.

## Declarations

### Registration

PROSPERO**—**CRD42024574365.
